# Hyaluronan Regulates Neuronal and Immune Function in the Rat Small Intestine and Colonic Microbiota after Ischemic/Reperfusion Injury

**DOI:** 10.3390/cells11213370

**Published:** 2022-10-25

**Authors:** Annalisa Bosi, Davide Banfi, Michela Bistoletti, Lucia Martina Catizzone, Anna Maria Chiaravalli, Paola Moretto, Elisabetta Moro, Evgenia Karousou, Manuela Viola, Maria Cecilia Giron, Francesca Crema, Carlo Rossetti, Giorgio Binelli, Alberto Passi, Davide Vigetti, Cristina Giaroni, Andreina Baj

**Affiliations:** 1 Department of Medicine and Surgery, University of Insubria, 21100 Varese, Italy; 2 Department of Pathology, Ospedale di Circolo, ASST-Sette Laghi, 21100 Varese, Italy; 3 Department of Internal Medicine and Therapeutics, Section of Pharmacology, University of Pavia, 27100 Pavia, Italy; 4 Department of Pharmaceutical and Pharmacological Sciences, University of Padova, 35131 Padova, Italy; 5 Department of Biotechnology and Life Sciences, University of Insubria, 21100 Varese, Italy

**Keywords:** intestinal ischemia/reperfusion injury, enteric nervous system, intestinal neuromuscular function, microbiota, TLRs

## Abstract

Background: Intestinal ischemia and reperfusion (IRI) injury induces acute and long-lasting damage to the neuromuscular compartment and dysmotility. This study aims to evaluate the pathogenetic role of hyaluronan (HA), a glycosaminoglycan component of the extracellular matrix, as a modulator of the enteric neuronal and immune function and of the colonic microbiota during in vivo IRI in the rat small intestine. Methods: mesenteric ischemia was induced in anesthetized adult male rats for 60 min, followed by 24 h reperfusion. Injured, sham-operated and non-injured animals were treated with the HA synthesis inhibitor, 4-methylumbelliferone (4-MU 25 mg/kg). Fecal microbiota composition was evaluated by Next Generation Sequencing. Neutrophil infiltration, HA homeostasis and toll like receptor (TLR2 and TLR4) expression in the small intestine were evaluated by immunohistochemical and biomolecular approaches (qRT-PCR and Western blotting). Neuromuscular responses were studied in vitro, in the absence and presence of the selective TLR2/4 inhibitor, Sparstolonin B (SsnB 10, 30 µM). Results: 4-MU significantly reduced IRI-induced enhancement of potentially harmful *Escherichia* and *Enterococcus* bacteria. After IRI, HA levels, neutrophil infiltration, and TLR2 and TLR4 expression were significantly enhanced in the *muscularis propria,* and were significantly reduced to baseline levels by 4-MU. In the injured, but not in the non-injured and sham-operated groups, SsnB reduced both electrical field-stimulated (EFS, 0.1–40 Hz) contractions and EFS-induced (10 Hz) non-cholinergic non-adrenergic relaxations. Conclusions: enhanced HA levels after intestinal IRI favors harmful bacteria overgrowth, increases neutrophil infiltration and promotes the upregulation of bacterial target receptors, TLR2 and TLR4, in the *muscularis propria*, inducing a pro-inflammatory state. TLR2 and TLR4 activation may, however, underlay a provisional benefit on excitatory and inhibitory neuronal pathways underlying peristalsis.

## 1. Introduction

Intestinal ischemia and reperfusion (IRI) injury is a life-threatening clinical condition and accounts for 0.1% of all hospital admissions [1,2]. Conditions leading to IRI include acute mesenteric arterial or venous thrombosis, embolism, intestinal transplantation, intestinal obstruction, trauma, shock and major surgery, neonatal necrotizing enterocolitis and chronic inflammatory bowel disease [3]. IRI causes severe damage to the intestine including transitory epithelial injury, increased vascular permeability and bacterial translocation [4,5]. Damage to the inner *muscularis propria* is long-lasting and affects muscle cells, enteric glia cells and neurons [4]. Myenteric neurons are particularly sensitive and undergo permanent damage, hampering digestion and transit [4,6]. IRI-induced neuronal injury may depend upon neuroimmune interactions caused by the recruitment of eosinophils and mast cells towards the *muscularis propria* and myenteric ganglia, which last for several weeks after IRI injury [4,7]. The gut microbiota may contribute to neuro-immune responses by releasing metabolites, i.e., damage associated molecular patterns, that activate toll like receptors located on both immunocytes and enteric neurons (TLRs, i.e., TLR2 and TLR4) [8,9,10,11].

The mechanism/s underlying this complex interplay are, however, still largely unknown and a better understanding of the microbiota–neuroimmune cross-talk in intestinal IRI injury may favor the development of innovative therapeutic approaches. Hyaluronan (HA), an unbranched glycosaminoglycan, may be involved in such interplay. HA is synthesized on cell surfaces as high molecular weight (HMW) polymers by HA synthases, namely, HAS1, HAS2 and HAS3 [12,13], with HAS2 being the most biologically active isoform in the gut neuromuscular compartment [7,14]. HMW-HA fragmentation into low molecular weight HA fragments (LMW-HA) favors immunocyte activation and cytokine release [15,16] as observed in the submucosal and *muscularis propria* layers of inflammatory bowel disease (IBD) patients and murine models of IBD [17,18]. HA may also modulate microbiota adhesion and translocation into the gut wall, and LMW-HA fragments act as damage associated molecular patterns, acting on TLR2 and TLR4 [19]. Recent evidence has shown that HA influences myenteric neuron architecture and function during both inflammation and IRI injury in the gut [7,14], and that HA deposition after IRI injury may influence neutrophil recruitment towards the *muscularis propria* and myenteric ganglia [7]. Interestingly, in spite of this pro-inflammatory effect, IRI-induced HA deposition in the *muscularis propria* and myenteric ganglia sustained gut transit efficiency by influencing excitatory and inhibitory myenteric plexus neurotransmitter pathways [7].

The goal of this study was to test the hypothesis that HA modulates the gut microbiota, nerve and immune response after in vivo IRI by altering molecular and morphological composition, affecting cellular functions in the small intestine. For this purpose, a rat model of superior mesenteric artery occlusion was used. The influence of HA on colonic microbiota composition was evaluated by Next Generation Sequencing of the bacterial 16S rRNA. HA deposition, neutrophil recruitment, and TLR2 and TLR4 expression were studied with and without treatment with 4-methylumbelliferone (4-MU), a blocker of HA synthesis [20]. One of the major physio-pharmacological effects of 4-MU is lowering HA production in different tissue types and cell lines [21]. The mechanism/s underlying 4-MU-induced inhibition of HA levels involve the reduction of an HA cytosolic precursor, UDP-glucuronic acid [22], and a not fully explained downregulation of HASes transcription [23]. The consequences of TLR2/4 activation on small intestine motor function were also assessed in vitro in the presence of a selective TLR2/4 antagonist, Sparstolonin B (SsnB) [24].

## 2. Materials and Methods

### 2.1. Animals and In Vivo Treatment

Male Wistar rats (Rattus Norvegicus, weight 250–350 g, age 49–63 days) were used for all experiments (animals were purchased from Envigo, San Pietro al Natisone, Udine, Italy). Animals had free access to a standard laboratory chow and tap water, and were maintained at a regular time-controlled 12/12 h light/dark cycle (light on at 8.00–20.00, temperature 22 ± 2 °C; relative humidity 60–70%), ensuring that lights were not used and that researchers and technicians did not enter the rat room during the dark cycle. Experimental groups consisted of injured animals undergoing ischemia/reperfusion injury (IRI), sham-operated animals undergoing laparotomy without IRI and non-injured rats, used as normal controls. Further experimental groups were represented by injured, sham-operated and non-injured treated with 4-methylumbelliferone (4-MU, 25 mg/kg i.p.) suspended in 25% DMSO in 0.9% NaCl, 24 h before euthanasia. This dosage was chosen considering its efficacy in downregulating HA production and the absence of adverse effect and toxicity, shown by previous studies [25]. The total number of animals used in the study was 90, and 15 animals were used in each experimental group. Animal care and handling were in accordance with ARRIVE guidelines and with the provisions of the European Union Council Directive 2010/63, recognized and adopted by the Italian Government (Decree No. 26/2014).

To induce IRI injury, rats were anesthetized with thiopental sodium (50 mg/kg) diluted (2% *w*/*v*) in sterile isotonic saline and given intraperitoneally (i.p.), in a non-fasted state as stated above. In some animals, one or two additional doses, consisting of 10% of the initial dose, were administered 20 min after the previous dose in order to maintain anesthesia. After laparotomy, a loop of the small intestine was exteriorized and a principal branch of the superior mesenteric artery (SMA) supplying the segment was temporarily occluded for 60 min with an atraumatic microvascular clamp, as described by Bistoletti et al., 2020 [7]. After recovery from anesthesia, animals were allowed access to food and water. Animals were euthanized by decapitation 24 h after reperfusion, when major histopathological (myenteric plexus and muscularis propria injury, neutrophilic infiltration), biomolecular (HIF1α, VEGFα and iNOS mRNA levels) and functional changes (slowing of the intestinal transit) have been observed [26]. Segments of the small intestine were immediately sampled from the region subjected to ischemia, discarding a 5 cm-long segment from the ileo-cecal junction, and rinsed with a physiological ice-cold Krebs solution [composition (mM): 118 NaCl, 4.7 KCl, 2.5 CaCl_2_∙2H_2_O, 1.2 MgSO_4_∙7H_2_O, 1.2 K_2_HPO_4_, 25 NaHCO_3_, 11 C_6_H_12_O_6_]. Whole-wall intestinal segments were fixed and stored for successive immunohistochemistry experiments. Quantification of HA levels, Western immunoblot and qRT-PCR studies were carried out using preparations consisting of external longitudinal muscle layer segments with attached myenteric plexus (LMMP) and submucosal layer, which included the submucosal plexus (SM), obtained immediately after excision of small intestine segments and stored at −80 °C.

### 2.2. Histology

Full-thickness small intestine samples were fixed in buffered formalin (4% *w*/*v* formaldehyde and acetate buffer 0.05 M) for 24–48 h and embedded in paraffin. Three-micrometer-thick sections were cut with a microtome and stained with Hematoxylin–Eosin (HE) for morphological analysis. Additional sections were mounted on poly-L-lysine-coated slides for immunohistochemical analysis of neutrophil infiltration, TLR2 and TLR4 staining. For myeloperoxidase (MPO) positive cell analysis, immunohistochemistry was performed with the avidin–biotin–peroxidase method using a polyclonal anti-MPO antiserum as described in Bistoletti et al. 2020 [7]. For TLR2 and TLR4 immunohistochemistry, cross-sections were incubated for 2 h in Tris-Buffered saline (TBS) containing 0.2% TritonX-100, 10% normal goat serum and 1% bovine serum albumin. Successively optimally diluted primary antisera (Table 1) were incubated overnight at 4° in Tris-Buffered saline (TBS) containing 0.2% TritonX-100 and 10% normal goat serum. Specific biotinylated secondary antibody and a peroxidase polymeric amplification system (HRP-Ultravision LP, LabVision, Suffolk, UK) were consecutively applied according to the manufacturer’s instructions and the reaction product was visualized with 3,3′-Diaminobenzidine (DAB). Sections were counterstained with hematoxylin, dehydrated and mounted with non-aqueous mounting medium (Sigma Aldrich 104095). Negative controls were obtained by omission of the primary antisera.

### 2.3. Immunohistochemistry

#### 2.3.1. Cross-Sections

Immunofluorescence experiments were carried out on 3 μm-thick intestinal sections embedded in paraffin [33]. Intestinal cross-sections were incubated in PBS with 2% bovine serum albumin (BSA) for 30 min and successively incubated overnight at 4 °C with a biotin-labeled HA binding protein (HABP; Hokudo, Sapporo, Japan). HABP binds to HA saccharides and localizes HA. Incubation with Fluorescein isothiocyanate-Streptavidin (FITC-Streptavidin) followed at RT for 1 h. Concentrations of HABP and FITC-Streptavidin are reported in Table 1. Negative controls were obtained by omission of either HAPB or FITC-Streptavidin. Coverslips were mounted using mounting medium with Vectashield-DAPI (Vector Laboratory, Burlingame, Canada). 

#### 2.3.2. Whole-Mount Immunohistochemistry

Segments of the rat small intestine were fixed in 4% formaldehyde with 0.2% picric acid in 0.2 mol/L phosphate buffer saline [PBS, composition (mM): 140 NaCl, 3 KCl, 15 Na_2_HPO_4_, 15 KH_2_PO_4_, pH 7.4], for 3–4 h at room temperature (RT), cleared of fixative and stored at 4 °C in PBS containing 0.05% thimerosal. Submucosal (SM) whole-mount preparations were obtained after removing the mucosal, *muscularis propria* and serosa layers, while LMMP whole-mount preparations were prepared according to the method described by Bistoletti et al., 2020 [34]. Submucosa and LMMP whole-mount preparations were incubated for 2 h with the same blocking PBS buffer containing 1% Triton X-100 (Sigma Aldrich, Merck Life Sci, Milan, Italy) and 10% normal horse serum (NHS) for nonspecific binding blocking (Celbio, Euroclone, Milan, Italy). Antisera buffer was the same for all samples and consisted of PBS buffer containing 1% Triton X-100 and 10% NHS. Double labeling was performed to evaluate HABP and HAS2 colocalization with HuC/D (pan-neuronal marker) in SM and LMMP preparations. To this end, the different primary antibodies and the related secondary antibodies were consecutively incubated at the optimal dilutions (Table 1). Preparations were mounted onto glass slides, using a mounting medium with DAPI (Vectashield; Vector Lab., Burlingame, CA, USA). 

### 2.4. Microscopy, Image Acquisition and Analysis

#### 2.4.1. Histology

The histological morphometric assessment was performed measuring eight different morphological criteria as described by Ceccotti et al., 2018 [33], consisting of villi height, width and density, crypt depth, submucosal layer thickness, total smooth muscle thickness, circular smooth muscle thickness and longitudinal smooth muscle thickness. Villi density was calculated as number of villi per 0.5 mm linear mucosa. The number of Goblet and Paneth cells per linear mucosal length (mm) was assessed. Cross-sections were stained for lysozyme to detect Paneth cells. Images were obtained with an Olympus IX51 optical microscope and investigated by a Fiji computer software ((https://imagej.net/software/fiji/publications (accessed on 1 November 2021). Measurements were repeated five times for each experimental group.

Neutrophil infiltration was evaluated in intestinal cross-sections by manually counting MPO^+^ cells in four high power fields (400×, diameter 0.55 mm). MPO value is reported as the average of MPO^+^ cells for field in each layer.

#### 2.4.2. Immunohistochemistry

Intestinal cross-sections and whole-mount preparations were analyzed by confocal microscopy with a Leica TCS SP5 confocal laser scanning system (Leica Microsystems GmbH, Wetzlar, Germany) and pictures were processed using Adobe Photoshop CS6.0 software.

HABP staining density index (i.e., pixel local density calculated as the number of pixels normalized by a unit area) in the mucosal, submucosal, *muscularis propria* and longitudinal muscle myenteric plexus (LMMP) layers was obtained by acquiring images with the same settings (gain, offset, exposure time and laser power, z-stacks number) within a short time frame. Uniform post-processing of intensity/contrast/brightness was performed. Ten fields per preparation (40× magnification) were analyzed using ImageJ software (version 1.52 t).

Immunoreactivity for HAS2 in intestinal whole-mount preparations was assessed by analyzing the density index of labeling per submucosal and myenteric ganglia area (10 fields per preparation at 40× magnification) as previously described [8]. All data were collected from at least five animals for each experimental group, employing a cohort size of 10–20 ganglia.

Abbreviations: Hyaluronan binding protein (HABP), Immunohistochemistry (IHC); Western Blot (WB). Supplying companies: AbClonal, Woburn, MA, USA; Abnova, Neihu District, Taipei City, Taiwan; Amersham, GE Healthcare, Buckinghamshire, UK; Cell Signaling Technology, Danvers, MA, USA; Dako, Glostrup Denmark; Hokudo Co., Ltd., Hokkaido, Japan; Invitrogen, Thermo Fisher Scientific, Paisley, UK; Jackson Immuno Research Laboratories, Baltimore, MD, USA; Molecular Probes, Thermo Fisher Scientific, Paisley, UK; Santa Cruz Biotechnology, Dallas, Texas, USA.

### 2.5. HA ELISA Assay

HA levels in mucosal, LMMP and SM preparations were evaluated using a Hyaluronan Quantikine ELISA Kit (R&D Systems, Minneapolis, MN, USA), following the manufacturer’s instructions. In brief, frozen mucosal, LMMPs and SMs obtained from 6 rats per group were lyophilized overnight and then suspended in cell lysis buffer provided by the kit overnight at RT, under gentle agitation. Debris were removed by centrifugation and supernatants were collected. Absorbance (Abs) values were recorded at 450 nm, with correction applied for optical imperfections in the plate performed by subtracting the Abs readings at 570 nm. HA levels were calculated as ng of HA per mg of dry tissue.

### 2.6. RNA Isolation and Quantitative RT PCR

Total RNA from small intestine SM and LMMP was extracted with TRIzol (Invitrogen) and treated with DNase I (DNase Free, Ambion), to remove possible traces of contaminating DNA. A quantity of 2 μg of total RNA was then retrotranscribed using the High-Capacity cDNA Synthesis Kit (Applied Biosystems, ThermoScientific, Waltham MA, USA) as previously described [34]. Quantitative RT_PCR (qRT-PCR) was performed with QuantStudioTM 3 Real-Time PCR System (Applied Biosystems, Merck Life Sci, Milan, Italy). mRNA levels for HAS2 (Rn00565774_m1) and the housekeeping gene β-actin (Rn00667869_m1) were evaluated with TaqMan Gene Expression Mastermix (Applied Biosystems). TLR2, TLR4 and β-actin mRNA levels were analyzed with Power Sybr Green Universal PCR Master Mix (Applied Biosystems). Primers were designed using Primer Express software (Applied Biosystems) (Table 2). Primer design was carried out to obtain a similar amplicon size and efficiency of amplification. The final concentration was 500 nmol/L for each primer. Relative gene expression was calculated with the 2^−ΔΔCt^ method. Experiments were replicated at least six times for each experimental group.

### 2.7. Western Immunoblot Analysis

Rat small intestine SM and LMMP segments were homogenized (Mixer Mill (MM301; Retsch GmbH, Hann, Germany)) for 30 min at 4 °C at 24,000 rpm with stainless steel beads (Next Advance, Troy, New York; NY, USA) in ice cold T-PER (ThermoScientific) containing a cocktail A with the following composition: 1 mM phenylmethylsulfonyl fluoride (PMSF), 10% protease inhibitor cocktail and 1µg/mL aprotinin (Sigma Aldrich). After sonication (4 × 4 s at 100% power) the homogenate was centrifuged at 12,000× *g* for 30 min at 4 °C. The supernatant was collected and incubated for 15 min at RT in a Tris-HCl 20 mM buffer containing cocktail A. Protein quantification was carried out with the Bradford method [35]. Sample dilution in Laemli buffer (Tris-HCl 300 mM, pH 6.8, glycerol 10%, SDS 2%, β-mercaptoethanol 0.04%) and protein denaturation for 5 min at 95 °C was then performed. Protein separation was carried out on 8% SDS-polyacrylamide gel electrophoresis (SDS-PAGE) and electroblotted to nitrocellulose membranes (Merck Millipore, Milan, Italy). Membranes were analyzed for TLR2 according to the protocol described in Bistoletti et al., 2020 [34], stripped and re-probed for TLR4 and β-actin as a loading control. Membrane stripping was performed by incubating with Tris/HCl 62.5 mM pH 6.8 with 2% SDS and 0.7% β-mercaptoethanol for 30 min at 60 °C, followed by 3 × 5 min washes in Tris-Buffered saline with 0.1% Tween-20 (TBS-T). Dilutions and main features of primary and secondary antisera are reported in Table 1. Bands were visualized by chemiluminescence (LiteAblot, Euroclone), acquired and quantified with Alliance Q9 Advanced System (Uvitec Ltd., Cambridge, UK). Experiments were performed at least six times for each experimental group. 

### 2.8. Next Generation Sequencing

Fecal pellets were collected in sterility in order to avoid possible environmental contamination and stored at −80 °C. On the day of sacrifice, animals were put in a sterile cage without bedding and were allowed to defecate. One fresh fecal pellet was collected with sterile forceps and put into a 1.5 mL sterile centrifuge tube and immediately frozen at −80 °C. Samples were then properly packaged and shipped to BMR Genomics (Padua, Italy) for microbiome analysis. Briefly, to extract genomic DNA, 100 mg fecal samples were suspended in 750 μL Bead Solution and 60 μL C1 solution (PowerFecal^®^, Qiagen, Düsseldorf, Germany) with 200–300 μL of zirconium-silica beads (0.1 mm; Biospec, Bartlesville, OK, USA) and incubated at 65 °C for 10 min. Samples were then vortexed in a TissueLyzer (Qiagen) for 10 min at 25 Hz and successively centrifuged at 13,000× *g* for 1 min. A quantity of 200 µL of the lysate was used for DNA extraction with Qiacube HT and Cador Pathogen 96 QIAcube HT Kit (Qiagen, Düsseldorf, Germany). A quantity of 100 µL of the obtained DNA was optimally diluted for amplification using specific primers with modification from Takahashi et al., 2014 [36] and tested on 1.5% agarose gel. Amplicons were purified with Agencourt XP 0.8X (Beckmann Coulter, CA, USA) magnetic beads and amplified with Index Nextera XT. Amplicons were then normalized with SequalPrep (ThermoFisher, Carlsbad, CA, USA) and sequenced in Illumina MiSeq (Illumina, San Diego, CA, USA) with V3 chemistry-300PE strategy. Raw sequences were processed using the software QIIME2 version 2019.4. Primer removal was performed using Cutadapt v4.1, while the reduction in background noise in the reading was performed using DADA2. This step included the review of the reading quality, the calculation of the error rate, any de-replications, the merging of the readings and the identification of chimeras. Each sample was then analyzed separately, and a taxonomic table was created using the OTU system at 99% from the Green Genes version 13-8 database. In order to determine the α and β diversity indexes, the Vegan R package was used. α index was calculated by different algorithms (Shannon, Simpson and Inverse Simpson), using the function “Diversity”; β index was calculated using the functions “Vegdist” and “Betadisper”. These indices were estimated on a database containing all the bacterial genus recognized by NGS. Alpha indices were then compared by one-way ANOVA followed by the Duncan post hoc test (Agricolae R package). Beta diversity analysis was performed by analysis of similarities (ANOSIM). For results display the GGPLOT2 R package was used.

### 2.9. Excitatory and Inhibitory In Vitro Motor Responses

After euthanasia, 2 segments of the small intestine (1 cm) were immediately excised 5 cm oral from the ileo-caecal junction, flushed with Krebs solution, cleared of connective tissue and mounted in isolated baths containing 10 mL of continuously oxygenated (95% O_2_ and 5% CO_2_) and heated (32 ± 1 °C) Krebs solution. Silk ligatures were applied to each end of the segment positioned along the longitudinal axis; one end was attached to a rigid support and the other to an isometric force displacement transducer (MDE Research GmbH, Walldorf, Germany). Mechanical activity was recorded with a PowerLab acquisition data system 8 (AD Instruments, UK) and analyzed with a LabChart 4.0 program (AD Instruments, UK). An initial load of 1 g was applied to each intestinal specimen. Tissues were allowed to equilibrate for 60 min prior to the start of the experiments. At the end of the experiments, intestinal segments were blotted on filter paper and weighed (g). To evaluate postjunctional cholinergic responses, for each segment, concentration–response curves to the muscarinic agonist, carbachol (CCh), were constructed cumulatively and plotted into a nonlinear regression model (fitted to a sigmoidal equation) to calculate EC_50_ and maximal tension (E_max_) values. Neuronally mediated contractions were obtained by Electric Field Stimulation (EFS, 5 and 10 Hz; 1 ms pulse duration, 10 s pulse train, 40 V) using platinum bipolar co-axial electrodes, attached to an MDE electronic stimulator (MDE Research). The effect of 5 and 10 Hz EFS was evaluated also in the presence of tetrodotoxin (TTX; 1 µM) to verify the neuronal origin of the response. TTX inactivates voltage dependent Na^+^ channels by directly binding to the pore forming α subunit of the channel, thereby preventing action potential generation and propagation [37].

Non-adrenergic non-cholinergic (NANC) on-relaxation and off-contractions, intestinal segments were stimulated at a frequency of 10 Hz after an incubation period of 20 min with atropine (1 μM) and guanethidine (1 μM). In order to evaluate the nitrergic inhibitory component of the on-relaxation, small intestine segments were incubated with L-Nω-Nitroarginine methyl ester chloridrate (L-NAME, a non-selective nitric oxide synthase (NOS) inhibitor, 100 μM) under NANC conditions. To evaluate the tachykinergic component of the small intestine contraction, the 10 Hz EFS-mediated off-contraction response was assessed in the presence of L-NAME, under NANC conditions. Under NANC conditions, the primary on-relaxation of small intestine segments was calculated as the AUC and normalized per g dry tissue weight to allow comparisons between tissue samples as described by Cerantola et al., 2020 [38]. Contractile off-responses were expressed as gram tension/gram dry tissue weight of small intestine segments. To evaluate the involvement of TLR2 and TLR4 receptors in the modulation of the neuromuscular layer of the rat small intestine, CCh-induced contractile responses, 5 and 10 Hz EFS responses, and 10 Hz EFS NANC responses were evaluated in the presence of the TLR2 and TLR4 antagonist Sparstolonin B at the concentrations of 10 and 30 µM. These concentrations were used on the basis of previously demonstrated in vitro efficacy [39].

### 2.10. Chemicals

All chemicals were obtained from Sigma–Aldrich (Milan, Italy), unless otherwise specified, and were of the highest commercially available analytical grade, with the lowest grade of 96%. All drugs for in vitro contractility studies were dissolved in milliQ water.

### 2.11. Statistical Analysis

Data are reported as mean ± standard error of the mean (SEM). Data obtained for the different variables displayed a normal distribution after the D’Agostino and Pearson normality test. Statistical significance was calculated by the unpaired Student’s *t*-test, by one-way ANOVA followed by Tukey’s post hoc test or by two-way ANOVA, when appropriate. Differences were considered statistically significant when *p* values were <0.05. For statistical analysis, the GraphPad Prism software was used (GraphPad 7.0 Software Inc., La Jolla, CA, USA).

## 3. Results

### 3.1. Effect of IRI Injury and HA Synthesis Blockade on Rat Small Intestine: General Observations and Histological Assessment

After occlusion of the terminal branch of the superior mesenteric artery, rat small intestine segments turned purple and returned to a normal pink color when normal blood flow was restored. Animals uneventfully recovered from anesthesia. Except for the scattered presence of hematomas on the surface, intestinal segments undergoing IRI did not show any gross anatomical anomalies compared to sham-operated and non-injured animals. After 4-MU treatment, intestinal segments and the mesenteric attachments appeared looser in all experimental groups, as compared to the respective untreated groups, although the inhibitor did not determine obvious morphological changes in intestinal segments obtained from all experimental groups.

**Figure 1:**
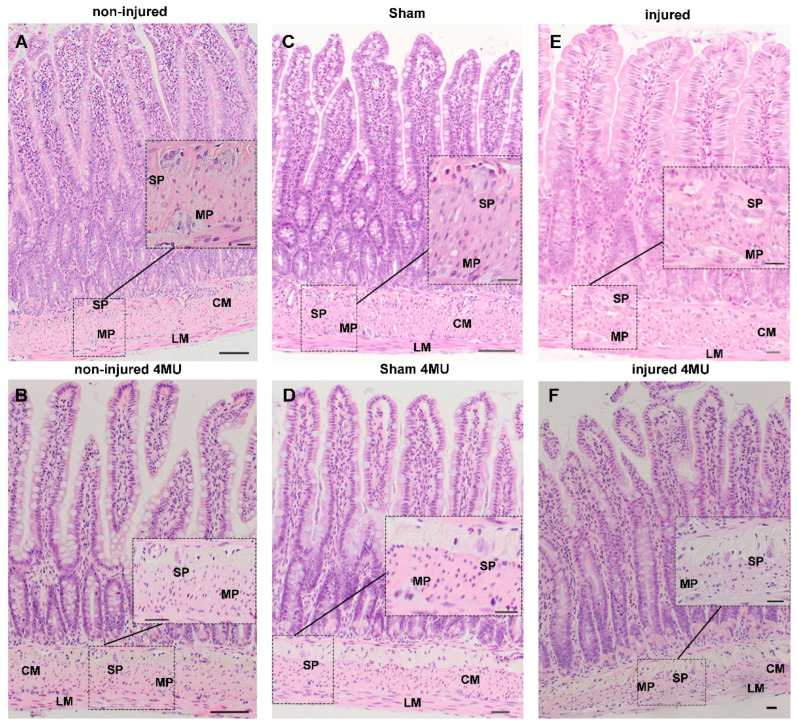
4-MU treatment prevents morphological changes in the rat small intestine wall after IRI. (**A**–**F**) Smooth muscle cells in the circular (CM) and longitudinal (LM) layers, submucosal (SP) and myenteric plexus (MP) of non-injured (**A**), non-injured 4-MU-treated (**B**), sham-operated (**C**), sham 4-MU-treated (**D**), injured (**E**) and injured 4-MU-treated (**F**) rat small intestine (Hematoxylin & Eosin, scale bar = 50 µm).

In the non-injured group, 4-MU treatment did not modify the architecture of the different intestinal layers or any of the morphometric parameters considered (Figure 1A,B; Supplementary Table S1).

4-MU treatment did not yield major histological changes in the mucosal and serosal epithelium of the sham-operated and injured groups (Figure 1C–F). The morphometric analysis of intestinal cross-sections (Supplementary Table S1) revealed that the mucosal layer thickness and villi height were significantly reduced in the injured group, when compared to non-injured (*p* < 0.01) and the sham-operated group (*p* < 0.001). The density of villi also increased when compared to the same groups (*p* < 0.01).

In the injured group, the mucosal thickness, and villi height and density, returned to values not significantly different from those of the non-injured group after 4-MU treatment. Crypt depth and the number of Goblet and Paneth cells did not significantly change after IRI. After 4-MU administration, a significant reduction in the number of Paneth cells was observed only in non-injured animals. 4-MU treatment did not influence crypt depth or the number of Goblet cells in all experimental groups (Supplementary Table S1).

After IRI, no major alterations of the submucosal layer, including the morphological features of submucosal ganglia, were observed. Furthermore, morphometric analysis of intestinal cross-sections revealed that the submucosal layer thickness was unaltered by injury and 4-MU treatment (Supplementary Table S1). After IRI injury, the smooth muscle layer displayed structural changes with vacuolated cytoplasm and appearance of spaces between cells (Figure 1E). However, such alterations did not influence the thickness of the circular and longitudinal layers, which was similar in all groups and was not influenced by 4-MU (Supplementary Table S1). In the injured group, myenteric neurons showed nuclear inclusions, swollen soma, vacuolated cytoplasm and irregular cellular membrane, suggesting an ongoing cellular damage (Figure 1E). After 4-MU treatment, in the *muscularis propria* and myenteric plexus, IRI-induced cytoplasmic vacuolization and spaces were significantly reduced (Figure 1F). In intestinal specimens obtained from sham-operated animals, histological features of both neuron and smooth muscle cellular injuries were rarely observed at the histological level and were completely absent after 4-MU (Figure 1C–D). Accordingly, the thickness of the circular and longitudinal smooth muscle layers remained unchanged with respect to values obtained in the non-injured group, and were unaltered in the 4-MU-treated group (Supplementary Table S1). 

### 3.2. Degree of Neutrophil Infiltration in the Different Intestinal Layers

**Figure 2:**
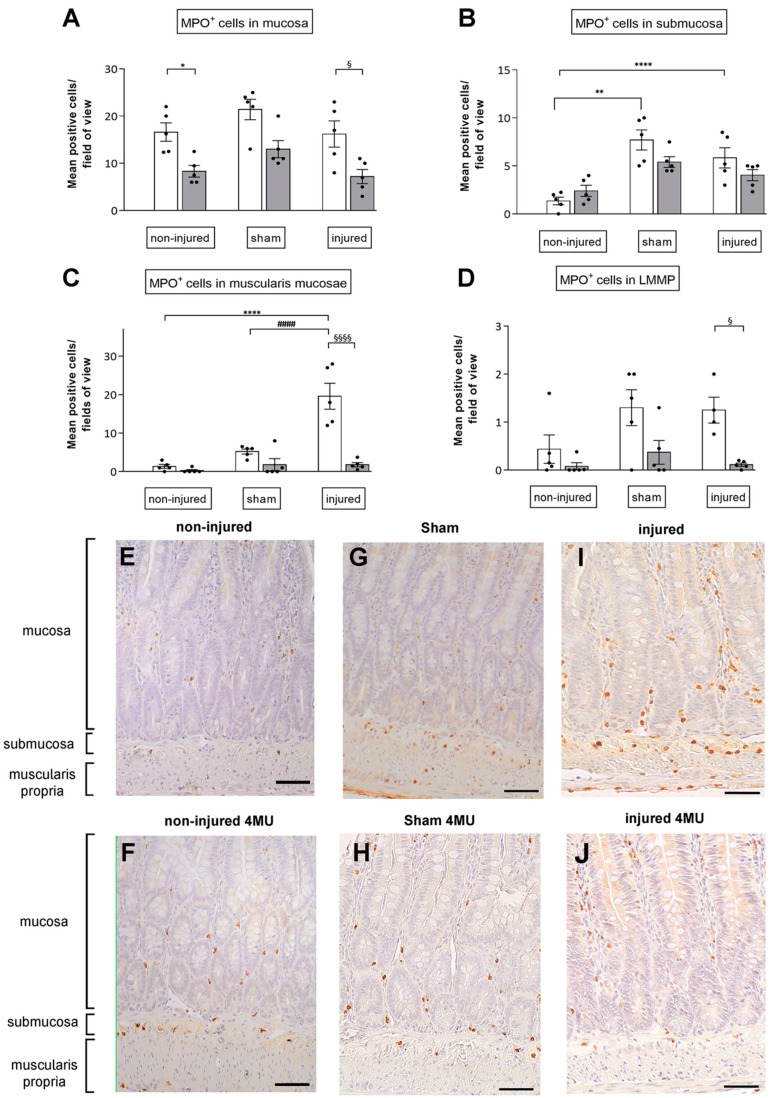
Immunostaining reveals increased number of MPO^+^ cells in the muscularis propria of injured animals. Neutrophil infiltrate expressed as number of MPO^+^ cells in the rat small intestine in the mucosa (**A**), submucosa (**B**), *muscularis propria* (**C**) and in myenteric ganglia (**D**) in the different experimental groups as indicated on top of graph in the absence (white columns) and presence of 4-MU 25 mg/kg (grey columns). Values are expressed as mean ± SEM of neutrophil count. ****^,§§§§,####^ *p* < 0.0001, ** *p* < 0.01, * *p* < 0.05 and ^§^ *p* < 0.05 by one-way ANOVA followed by Tukey’s test. N = 5 rats/group. (**E**–**J**): MPO^+^ cells are evident for their brown staining (panels are representative of cross-sections obtained from all experimental groups as indicated, scale bar = 20 µm).

Myeloperoxidase (MPO) immunohistochemical staining revealed a similar number of neutrophils per field in the mucosal layer of non-injured, sham-operated and injured animals (Figure 2A,E,G,I). 4-MU induced a reduction in neutrophil infiltration in the mucosa in all groups, which was statistically significant in the non-injured and injured groups (Figure 2A,F,H,J). In the submucosal layer of sham-operated and injured groups, the number of neutrophils significantly increased with respect to the non-injured (*p* < 0.0001 and *p* < 0.01, respectively; Figure 2B,E,G,I), and was unchanged by 4-MU (Figure 2B,F,H,I). In the *muscularis propria*, the number of MPO+ cells per field significantly increased in the injured (*p* < 0.0001) with respect to non-injured and sham-operated groups (Figure 2C,E,G,I). IRI-induced neutrophil infiltration in the *muscularis propria* was significantly reduced after 4-MU treatment (*p* < 0.0001) (Figure 2C,F,H,K). 4-MU treatment reduced, although not significantly, neutrophilic infiltration in the *muscularis propria* both in non-injured and sham-operated groups (Figure 2C,F,H,K). 4-MU reduced the number of neutrophils infiltrating myenteric ganglia in all groups, which was statistically significant in the non-injured and injured, but not in the and sham-operated group (Figure 2D–L). Overall, these results suggest that 4-MU shows protective properties in the *muscularis propria* of injured animals against neutrophilic infiltration. In addition, in the mucosa, *muscularis propria* and myenteric ganglia, 4-MU influences neutrophilic recruitment in all experimental conditions. 

### 3.3. Effect of IRI Injury and HA Synthesis Blockade in Rat Small Intestine 

**Figure 3:**
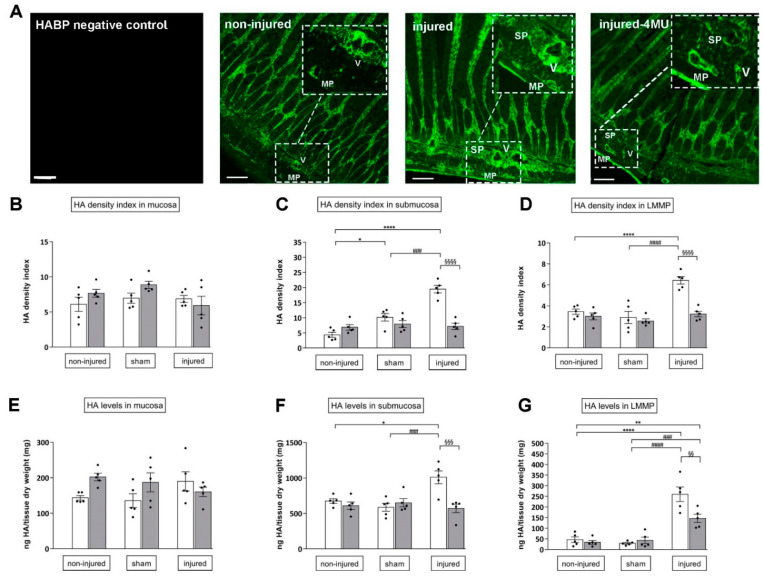
Immunofluorescence staining and ELISA assay reveal enhanced HA levels in the submucosa and LMMPs after IRI. (**A**) Confocal microphotographs of HA staining in rat small intestine cross-sections obtained from a negative control, non-injured, non-treated injured and 4-MU-treated injured groups (V: vessel; MP: myenteric plexus; SP: submucosal plexus). Scale bar 100 µm. (**B**–**D**): HA density index in the mucosa (**B**), submucosa (**C**) and longitudinal muscle with the myenteric plexus (LMMP) preparations (**D**) obtained in non-injured, sham and injured groups with (grey columns) or without (white columns) 4-MU treatment. (**E**–**G**) HA levels quantified with ELISA assay in rat small intestine mucosa (**E**), submucosa (**F**) and LMMP preparations (**G**), obtained from the different experimental groups, with (grey columns) or without (white columns) 4-MU. HA levels expressed as ng of HA normalized per mg of dry tissue weight. Data are reported as mean ± SEM. N = 5 rat/group. ****^,§§§§,####^ *p* < 0.0001, ^###,§§§^ *p* < 0.001, **^,§§^ *p* < 0.01, * *p* < 0.05 by one-way ANOVA with Tukey’s post hoc test.

In cross-section of non-injured rat small intestine, HA staining was found in the *lamina propria mucosae* of the epithelium. In the submucosal layer, a particularly remarkable HA labeling was found in the tunica media and adventitia of large and medium-sized vascular vessels. A fainter HA labeling was found in the longitudinal muscle and myenteric plexus (LMMP) with respect to the rest of the intestinal wall (Figure 3A). HA staining in the different layers of non-injured intestinal preparations reflected values of HA density index (Figure 3B–D) and HA levels (Figure 3E–G). In sham-operated animals, HA density index significantly increased in the submucosal layer with respect to values obtained in non-injured animals (*p* < 0.05), but remained unchanged in the mucosa and in LMMPs (Figure 3A,C). These data were confirmed by ELISA assay, except for HA density index enhancement in the submucosal layer (Figure 3E–G). In both non-injured and sham-operated groups, 4-MU treatment did not significantly influence either HA density index or HA levels (Figure 3B–G). In injured animals, HA density index and levels in the submucosal layer and LMMPs significantly increased with respect to both the non-injured and sham-operated group (density index: *p* < 0.0001 and *p* < 0.01 and HA levels: *p* < 0.05 and *p* < 0.001, respectively) (Figure 3C–G), and remained unchanged in the mucosa layer (Figure 3A,B,E). In the submucosal layer and LMMPs, 4-MU significantly reduced IRI-induced enhancement of both density index and HA levels (*p* < 0.001) (Figure 3C–G). 

### 3.4. Effect of IRI Injury and HA Synthesis Blockade in Rat Small Intestine Submucosal Ganglia

**Figure 4:**
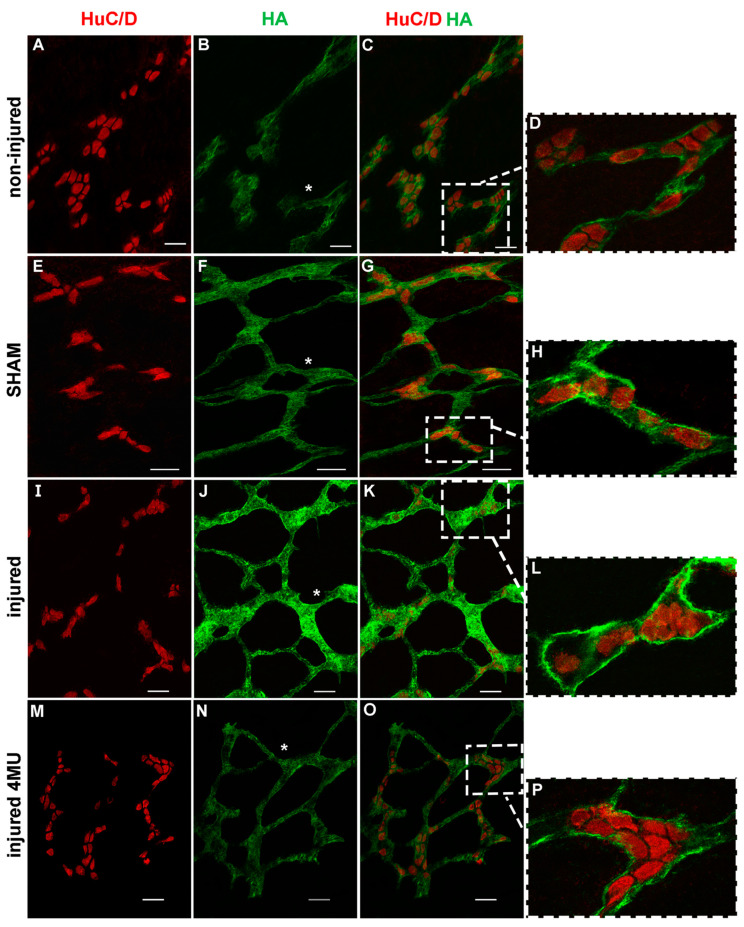
HA immunofluorescence staining increases in rat small intestine submucosal plexus after IRI injury and is reduced after MU treatment. (**A**–**P**) Confocal microphotographs of HA staining in the rat small intestine submucosal plexus in non-injured group (**A**–**C**); (**D**) median section at higher magnification of panel (**C**), sham-operated group (**E**–**G**); (**H**) median section at higher magnification of panel (**G**), untreated injured group (**I**–**K**); (**L**) median section at higher magnification of panel (**K**) and in the 4-MU-treated injured group ((**M**–**P**); (**R**) median section at higher magnification of panel (**Q**) groups)). In all groups, HA stained the surface of submucosal plexus and interconnecting fibers (*) (panels (**B**,**F**,**J**,**N**)). In submucosal ganglia median sections, HA immunofluorescence was prevalently found in the perineuronal space and in the neuron soma (panels (**D**,**H**,**L**,**P**)), as demonstrated by double-staining with the pan-neuronal marker, HuC/D. Microphotographs were obtained with the same gain resolution. Scale bar = 50 µm. N = 5 rat/group.

In whole-mount preparations of the rat small intestine submucosa, HA staining was found on the surface of submucosal ganglia and along interconnecting fibers (Figure 4A–P). In addition, co-localization with the pan-neuronal marker HuC/D demonstrated a faint HA labeling in submucosal neuron cytoplasm and a more intense labeling in the perineuronal space (Figure 4D,H,L,P). In preparations obtained from injured animals, HA staining was enhanced on ganglia surfaces, in neuron cytoplasm and in the perineuronal space if compared to non-injured and sham-operated groups (Figure 4I–K), and was attenuated by 4-MU treatment (Figure 4M–O).

**Figure 5:**
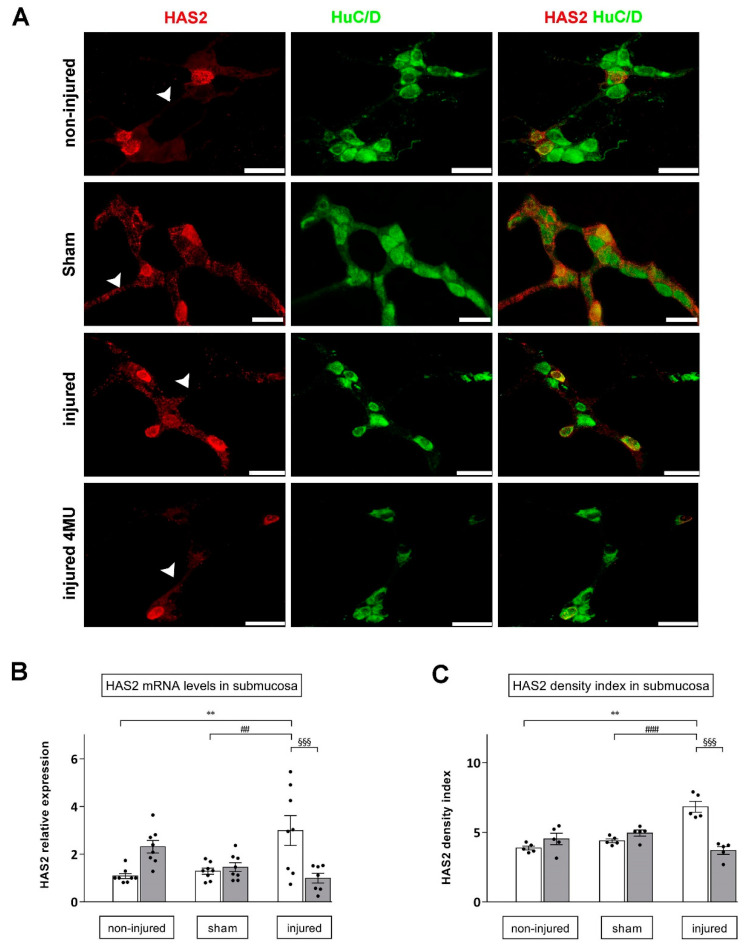
Immunofluorescence staining and qRT-PCR reveal increased HAS2 expression in the rat small intestine submucosal plexus after IRI injury. (**A**) Confocal microphotographs of HAS2 and HuC/D co-localization in submucosal neurons of CTR, sham-operated animals, after IRI injury and in the 4-MU-treated injured group. HAS2 stained the soma of ovoid submucosal neurons. Scale bar = 50 µm. (**B**) HAS2 mRNA levels in submucosal layer obtained from the different experimental groups, with (grey columns) or without (white columns) 4-MU treatment. Graphs show HAS2 relative gene expression to β-actin. ^§§§^ *p* < 0.001, ** *p* < 0.01, ^##^ *p* < 0.01 by one-way ANOVA with Tukey’s test. N = 6 rat/group. (**C**) Density index of HAS2 staining in the submucosal plexus obtained from the different experimental groups, with (grey columns) or without (white columns) 4-MU treatment. ^###,§§§^ *p* < 0.001, ** *p* < 0.01, *p* < 0.0001 by one-way ANOVA with Tukey’s test. N = 5 rat/group.

In the submucosal layer whole-mount preparations obtained from non-injured animals, HAS2 staining was observed in the cytoplasm, nuclear and cytosolic membrane of a few large and medium ovoid neurons and along interconnecting fibers (Figure 5A). In the submucosal layer, HAS2 mRNA levels significantly increased after IRI with respect to both non-injured and sham-operated preparations (*p* < 0.01) (Figure 5C). Such enhancement was significantly reduced by 4-MU treatment (*p* < 0.001 vs. IRI). Accordingly, in the submucosal plexus of the injured group, the density index of HAS2 staining was significantly higher with respect to values obtained in the non-injured and sham-operated groups (*p* < 0.0001) (Figure 5C). Such enhancement was significantly reduced after 4-MU treatment with respect to the relative untreated injured group (*p* < 0.001) (Figure 5C). 4-MU treatment did not modify HAS2 density index and mRNA expression in both non-injured and sham-operated preparations (Figure 5B,C). 

### 3.5. 4-MU Influences TLR2, TLR4 Expression of in the Rat Small Intestine after Iri Injury

**Figure 6:**
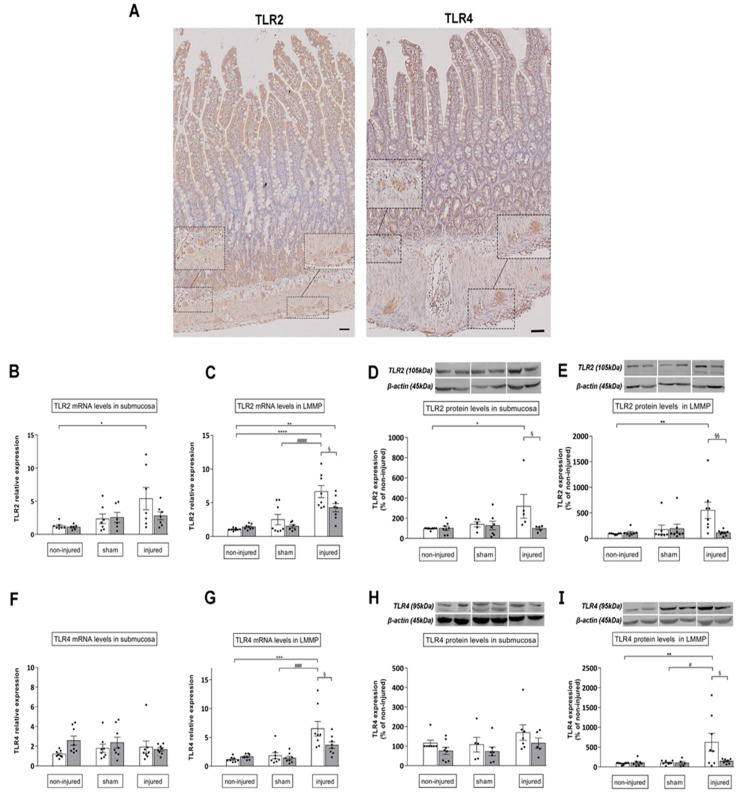
Histochemical, qRT-PCR and Western blotting disclose IRI injury-induced enhancement of TLR2 and TLR4 expression in the rat small intestine. (**A**) Distribution of TLR2 and TLR4 in rat small intestine cross-sections in non-injured conditions. Scale bar = 50 µm. (**B**–**E**) Relative expression of mRNA and protein levels of TLR2 (**B**,**C**) and TLR4 (**F**–**I**) in the submucosal layer and LMMP preparations obtained from the different experimental groups, with (grey columns) or without (white columns) 4-MU treatment. TLR2 and TLR4 relative gene expression was determined by calculating 2^−ΔΔCt^ values normalized to β-actin, and ****^,####^ *p* < 0.0001, ***^,###^ *p* < 0.001, ** *p* < 0.01, *,^§^ *p* < 0.05 by one-way ANOVA with Tukey’s post hoc test. Values are expressed as mean ± SEM, n = 6–8. For TLR2 and TLR4 protein expression levels, blots representative of immunoreactive bands for TLR2, TLR4 and β-actin in the different experimental conditions are reported on top of each panel. Samples (200 µg) were electrophoresed in SDS-8% polyacrylamide gels. Numbers at the margins of the blots indicate relative molecular weights of the respective protein in kDa. Data are expressed as mean ± SEM (n = 6–8) of the optical density (O.D.) ratio of TLR2 and TLR4 vs. β-actin in the different experimental conditions normalized to values obtained in the non-injured group. **^,§§^ *p* < 0.01, *^,#,§^ *p* < 0.05 by one-way ANOVA with Tukey’s post hoc test.

In control preparations, TLR2 immunoreactivity was detected in all layers of the gastrointestinal wall. TLR2 antiserum stained the cytoplasm of enteric epithelial cells with a major intensity in the crypt compartment and epithelial surface. TLR2 immunoreactivity was also observed in neurons and glial cells in submucosal and myenteric ganglia and in smooth muscle cells (Figure 6A). Rare inflammatory cells were also observed along the whole wall. TLR4 antiserum weakly stained epithelial cells located in the crypts and at the top of villi, and some disperse inflammatory cells. The presence of TLR4 was also evidenced in some neurons and in glial cells of both the submucosal and myenteric ganglia (Figure 6A). 

In the submucosal layer after IRI, both TLR2 mRNA and protein expression levels significantly increased with respect to the control group (*p* < 0.05) (Figure 6B). In this condition, only TLR2 protein levels were significantly reduced after 4-MU administration (*p* < 0.05) (Figure 6D). In rat small intestine LMMP preparations of injured animals, TLR2 mRNA levels were significantly higher with respect to both non-injured and sham-operated groups (*p* < 0.001) (Figure 6C), while TLR2 protein levels significantly increased only with respect to values obtained in the non-injured group (*p* < 0.01) (Figure 6E). Both transcript and protein TLR2 levels were significantly reduced in LMMPs obtained from 4-MU-treated injured animals, with respect to the untreated injured group (*p* < 0.05 and *p* < 0.01, respectively). In the submucosa and LMMPs of non-injured and sham-operated animals, TLR2 mRNA and protein expression levels were similar, in the absence and presence of 4-MU (Figure 6B–E). 

In the submucosal layer of all experimental groups, TLR4 mRNA and protein expression levels were similar (Figure 6F,H). In LMMP preparations of injured animals, TLR4 mRNA and protein levels were significantly higher than in the non-injured and sham-operated groups (*p* < 0.001, *p* < 0.01 and *p* < 0.05, respectively) (Figure 6G,I). In LMMP preparations obtained from the 4-MU-treated injured group, both transcript and protein levels of TLR4 receptors were significantly reduced with respect to the untreated injured group (*p* < 0.05) (Figure 6G,I). TLR4 mRNA and protein expression level were not significantly different in LMMP preparations obtained from sham-operated and non-injured animals and were not affected by 4-MU treatment (Figure 6F–I).

### 3.6. Gut Microbiota Composition

**Figure 7:**
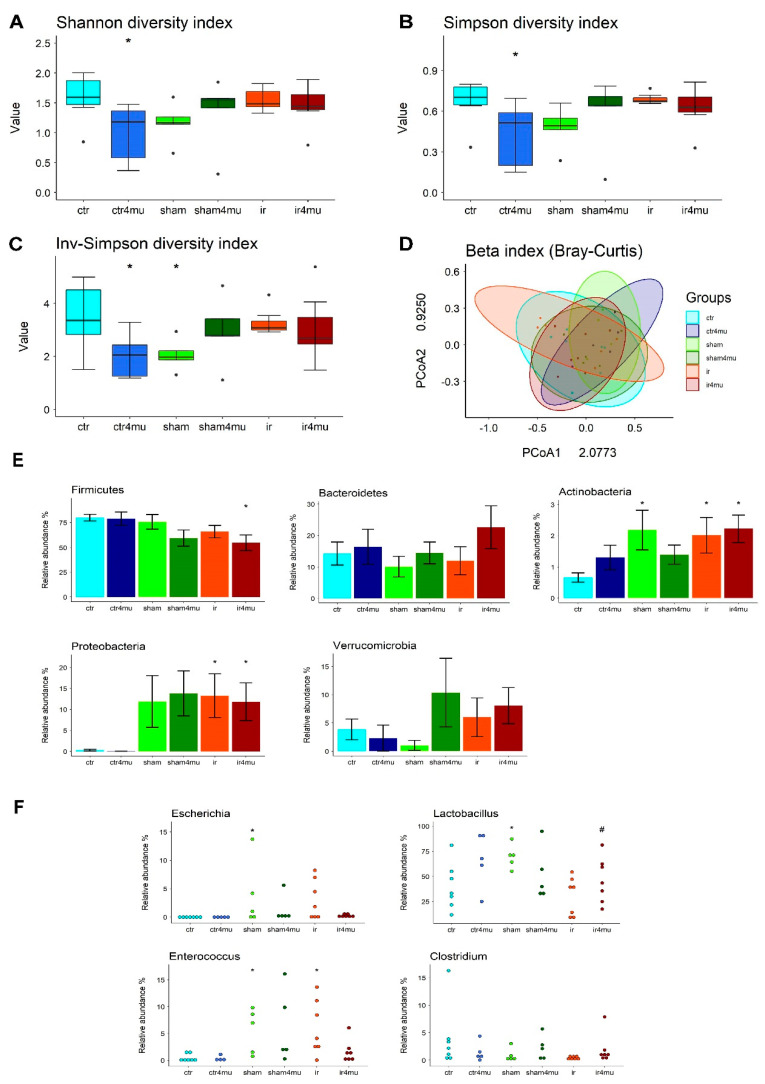
4—MU treatment alters the composition of bacterial gut microbiome after IRI injury. (**A**–**C**) Estimation of microbial community α-diversity by Shannon, Simpson and inverse-Simpson indexes. Black dots represent the outliers. * *p* < 0.05, vs control group by ANOVA with Duncan post hoc test. (**D**) Principal Coordinates Analysis (PCoA) plot illustrating gut microbiome β-diversity calculated using the Bray–Curtis dissimilarity. After analysis of similarities (ANOSIM), no significant differences were observed among the different experimental groups. Values are expressed as mean ± SEM. * *p* < 0.05 by ANOVA with Duncan’s post hoc test with respect to non-injured. (**E**) Relative abundance, expressed as percentage of total sequences of selected phylum and genus (**F**), identified by NGS of the bacterial 16S mRNA in the different experimental groups. Values are expressed as mean ± SEM. * *p* < 0.05 by ANOVA with Duncan’s post hoc test with respect to non-injured; ^#^ *p* < 0.05 by ANOVA with Duncan’s post hoc test with respect to injured.

Since glycosaminoglycans have important effects on the gut microbiota homeostasis, we first assessed if HA synthesis blockade by 4-MU could influence the composition of the bacterial saprophytic community 24 h after the mesenteric ischemic injury. Analysis of fecal samples’ NGS sequencing in the V3–V4 hypervariable region of prokaryotic 16S rRNA was performed by calculating α and β diversity indices, representing the variability of gut microbiota composition at the single sample level and community level, respectively. As shown in Figure 7A–C, IRI injury was not associated with significant changes in the diversity of the microbial community, nor did 4-MU influence this parameter in the IRI group, as measured by Shannon, Simpson and inverse Simpson α indices. In the sham-operated group a reduced diversity was evidenced, which was statistically significant for the inverse Simpson’s α index; Figure 7A–C. This reduction was not observed after 4-MU treatment. In the non-injured group, treatment with 4-MU significantly reduced the microbial diversity for all indices.

No significant differences were observed among the microbial communities in the different experimental groups by calculating β diversity, using the Bray–Curtis dissimilarity approach (Figure 7). Possible variations in microbiota composition were also analyzed at the phylum level. In the injured and 4-MU-treated injured groups, the abundance of the Firmicutes phylum was lower than in the non-injured group, reaching a statistical significance in the 4-MU injured group (Figure 7E). A reduced abundance of Firmicutes was also observed in the sham-operated group after 4-MU treatment. A significant increase in the relative abundance of Proteobacteria was observed in the untreated and 4-MU-treated injured groups, as compared to non-injured animals (Figure 7E). Moreover, in the untreated and 4-MU-treated sham-operated groups, Proteobacteria levels increased with respect to non-injured animals. 4-MU treatment did not modify Proteobacteria levels in fecal samples obtained from non-injured animals. The relative abundance of Actinobacteria significantly increased in the injured and 4-MU-treated injured groups and after sham operation (Figure 7E). No significant differences in the relative abundance of Bacteroidetes and Verrucomicrobia were noticed among the different experimental groups, (Figure 7E). An increase in *Escherichia* genus abundance was observed in the injured and sham-operated (*p* < 0.05 only in sham-operated) groups with respect to values obtained in samples obtained from non-injured animals (Figure 7F). In both experimental groups, 4-MU treatment induced a decrease in the relative abundance of *Escherichia*, which was more remarkable in the injured group (Figure 7F). The abundance in the *Enterococcus* genus significantly increased in the injured and sham-operated groups (*p* < 0.05; Figure 7F). This enhancement was significantly reduced only in the injured group after 4-MU treatment (Figure 7F). In the injured group, the abundance of *Lactobacillus* and *Clostridium* genus was reduced with respect to the other groups and was partially restored towards control values by 4-MU (Figure 7F). No significant changes were observed for the genus *Clostridium* in the sham-operated group, with or without 4-MU treatment, with respect to values obtained in non-injured animals. In the sham-operated group, *Lactobacillus* levels were significantly higher than non-injured values and were not significantly modified by 4-MU treatment. In the non-injured group, 4-MU treatment did not modify the relative abundance of *Escherichia*, *Enterococcus*, *Lactobacillus* and *Clostridium* genera (Figure 7F).

### 3.7. Effect of the TLR2 and TLR4 Inhibitor Sparstolonin B on the Rat Small Intestine Neuromuscular Function after IRI Injury

The possible involvement of TLR2 and TLR4 in the modulation of the small intestine neuromuscular function after an IRI injury was investigated by measuring in vitro the effect of the TLR2 and TLR4 inhibitor Sparstolonin B (SsnB) on excitatory contractions and inhibitory relaxations of the rat small intestine longitudinal muscle. In all experimental groups, intestinal specimens displayed spontaneous basal activity consisting of phasic pendular contractions. In the injured group with or without SsnB (10 and 30 µM), the amplitude (tension) of spontaneous contractions was significantly reduced with respect to non-injured and sham-operated animals (*p* < 0.001 for both the experimental groups) (Supplementary Figure S1). In the injured group, the frequency of spontaneous basal contractions was significantly higher compared to controls, and was not influenced by addition of SsnB (Supplementary Figure S1). In the sham-operated group, both the amplitude and frequency of spontaneous contractions were not significantly different with respect to values obtained in non-injured animals, with or without SsnB.

**Figure 8:**
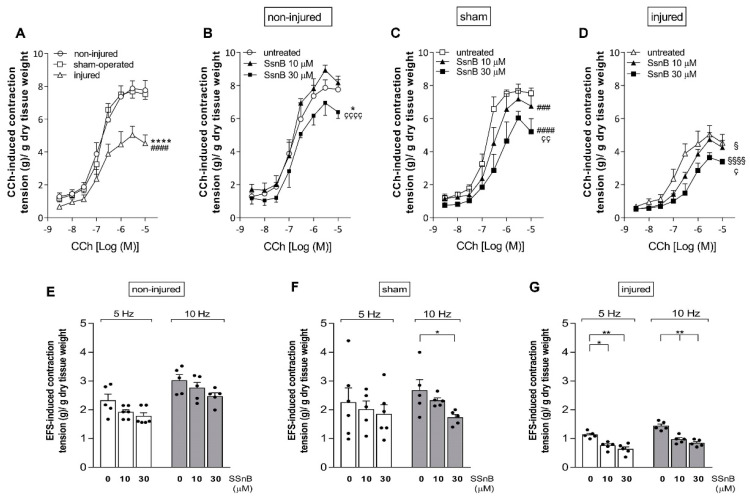
Blockade of TLR2/4 receptors reduces carbachol—and EFS—induced contractions of the rat small intestine after IRI injury. (**A**–**D**) Concentration–response curves to carbachol (CCh) of the rat small intestine longitudinal muscle in the absence and presence of Sparstolonin B (SsnB) 10 and 30 µM (N = 5 rats per group). * *p* < 0.05, **** *p* < 0.001 vs non-injured; ^###^ *p* < 0.001 ^####^ *p* < 0.0001 vs. sham-operated; ^§^ *p* < 0.05, ^§§§§^ *p* < 0.0001 vs. injured; ^ç^ P < 0.05; ^çç^ *p* < 0.01 and ^çççç^ *p* < 0.0001 vs. SsnB 10 µM in the respective group by Two-way ANOVA with Tukey’s test (**A**,**B**). (**E**–**G**) Excitatory responses induced by EFS at 5 Hz (white columns) and at 10 Hz (grey columns) in the rat small intestine longitudinal muscle of non-injured (**E**), sham-operated (**F**) and injured (**G**) groups in the absence and presence of SsnB 10 µM and 30 µM as indicated on the bottom of graphs. Data are reported as mean ± SEM. N = 5 rat/group. Statistical significance: * *p* < 0.05, ** *p* < 0.01 by one-way ANOVA with Tukey’s post hoc test.

To investigate changes in the excitatory neuromuscular response, cumulative concentration–response curves to the non-selective cholinergic agonist, carbachol (CCh), were performed on longitudinally oriented small intestine segments from all experimental groups. IRI induced a significant downward shift of the concentration–response curve to CCh with a decrease in maximum response (E_max_) compared to values obtained from non-injured and sham-operated preparations (*p* < 0.001 for both experimental groups) (Figure 8A).

SsnB induced a significant dose-dependent downward shift of the concentration–response curve to CCh both in the injured (SsnB 10 µM: *p* < 0.05; SsnB 30 µM: *p* < 0.0001, respectively) and sham-operated (SsnB 10 µM: *p* < 0.001; SsnB 30 µM: *p* < 0.0001, respectively) groups (Figure 8C–D). In the non-injured group, SsnB decreased the E_max_ of the CCh concentration–response curve only at the higher concentration tested (SsnB 30 µM: *p* < 0.05 with respect to non-injured values, and *p* < 0.0001 with respect to the curve in the presence of SsnB 10 µM). SsnB did not influence the potency of CCh concentration–response curves as suggested by the similar EC_50_ values obtained in the different experimental groups, with or without SsnB at both concentrations tested (Supplementary Table S2).

To further investigate potential changes in the excitatory contractile function, the effect of EFS was evaluated at increasing frequencies of stimulation of rat small intestine longitudinal muscle. In the injured group, EFS-induced contractions at both 5 and 10 Hz were significantly reduced compared to non-injured and sham-operated groups (data not shown). SsnB at 10 and 30 µM induced a further significant decrease of EFS-induced contractions both at 5 Hz (10 µM *p* < 0.05 and *p* < 0.001 30 µM) and 10 Hz (*p* < 0.001 for both SsnB concentrations) (Figure 8G). In non-injured and sham-operated groups, EFS-induced contractions were not significantly reduced by SsnB either at 5 Hz or at 10 Hz, except for a significant reduction in EFS-induced stimulation at 10 Hz in the sham-operated group induced by 30 µM SsnB (*p* < 0.05) (Figure 8E–F). EFS-mediated responses were totally abolished by addition of the neuronal blocker TTX (1 µM), suggesting their neuronal origin.

**Figure 9:**
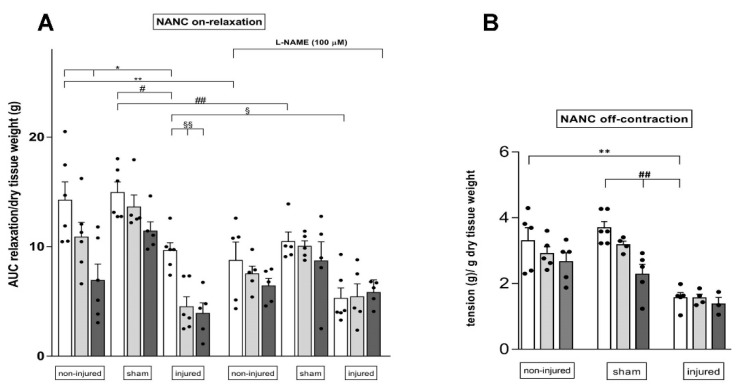
Blockade of TLR2/4 receptors reduces NANC on-relaxations and does not influence off-contractions in the rat small intestine after IRI injury. (**A**) Ten hertz EFS-evoked NANC on-relaxation responses of the rat small intestine longitudinal muscle in non-injured, sham-operated and injured groups in the absence (white columns) and presence of Sparstolonin B (SsnB) 10 µM (light grey columns) and 30 µM (dark grey columns). Some experiments were conducted in the presence of L-NAME 100 µM. (**B**) Tachykinergic-mediated contractions evoked by 10 Hz EFS in small intestine segments under NANC conditions, in the presence of L-NAME. Data are reported as mean ± SEM. N = 5 rats/group. Statistical significance: *^,#,§^ *p* < 0.05, **^,##,§§^ *p* < 0.01 by one-way ANOVA followed by Tukey’s post hoc test.

NANC small intestine EFS-induced on-relaxations, measured at 10 Hz in the presence of guanethidine and atropine, were also influenced by IRI. As shown in Panel A of Figure 9, in the injured group, NANC relaxations were significantly reduced with respect to the inhibitory responses obtained in non-injured and sham-operated animals (*p* < 0.05 against both groups). In the injured group, both 10 and 30 µM SsnB significantly reduced the relaxation response (*p* < 0.01) (Figure 9A).

In the non-injured and sham-operated groups, SsnB did not significantly influence NANC relaxations, except for the significant decrease observed in the presence of 30 µM SsnB in the non-injured group (Figure 9A). In all experimental groups, addition of the nitric oxide synthase inhibitor L-NAME (100 µM) significantly reduced the relaxation response. At all tested concentrations, SsnB did not influence intestinal relaxation of intestinal segments in the presence of L-NAME (Figure 9A). At the end of the on-relaxation, EFS stimulation in NANC conditions induces an off-contraction, which is mainly of tachykinergic origin. In the injured group, the rebound NANC contraction at 10 Hz EFS stimulation, measured in the presence of L-NAME, was significantly lower than the contractile response measured in non-injured and sham-operated groups (Figure 9B). After IRI, SsnB, at 10 and 30 µM did not influence NANC off-contractions. In the non-injured and sham-operated groups SsnB, did not significantly influence rebound contractions, except for a significant decrease observed in the presence of 30 µM SsnB in the sham-operated group (Figure 9B).

## 4. Discussion

This study demonstrates that HA is involved in the modulation of neuronal and immune function and of gut microbiota after in vivo IRI injury in the rat small intestine. After IRI, HA deposition in the small intestine wall increases from the submucosa to the *muscularis propria.* Furthermore, HA influences neutrophil recruitment and the expression of TLR2 and TLR4 transcript and protein, in the *muscularis propria* after IRI. In this condition, activation of TLR2/TLR4 provisionally contributes to sustain the gut motor function. After IRI in the small intestine, HA may also influence the composition of the colonic luminal flora, which hosts the most complex and abundant microbial community in the gut [40]. 

After IRI injury, HA levels were upregulated in the submucosal layer and in the muscularis externa of the rat small intestine and remained unchanged in the mucosal layer, which is the first layer to undergo restitutio ad integrum. According to other studies, twenty-four hours after temporary occlusion of the superior mesenteric artery, the epithelium did not display major signs of damage [4,41], although a more accurate morphometric analysis showed shorter villi, as already demonstrated in the mouse ileum in the same IRI conditions [41], suggesting incomplete villi reconstitution. Interestingly, epithelial morphometric parameters similar to those obtained in non-injured animals were found in the IRI group after 4-MU treatment, indicating that IRI-induced HA de novo synthesis may influence epithelial cell turnover.

As already observed in mouse models of IBD and in IBD patients, in our rat model, the highest level of IRI-induced HA deposition was observed in the submucosal layer, as suggested by the intense HA staining both in blood vessels and submucosal ganglia [18]. We cannot exclude that IRI-induced HA deposition in blood vessels plays a role in leukocyte recruitment, as demonstrated during gut inflammation [18]. Indeed, HA is recognized as a fundamental regulator of vascular cell homeostasis in health and disease conditions [42] and may participate in IRI-induced permeability changes [43]. However, in the injured group, we could not find significant histological changes in the submucosal layer, excluding an overt inflammatory injury with formation of fibrotic tissue, as occurs in IBD [18,44]. The absence of significant alterations in the mucosa and submucosa may represent an IRI-induced regenerative response attenuating the development of more vigorous inflammatory response, as suggested to occur after IRI injury in the human small intestine [45]. However, our data reflect the limited translational value of our model for the study of the pathogenesis of IBD, which is characterized by mucosal barrier dysfunction, leading to an exaggerated response to commensal microbiota and loss of intestinal immune homeostasis [46].

HA is synthesized in submucosal ganglia, concurring to the formation of the ganglia basal lamina and of a perineuronal net, as already described in the rat myenteric plexus [7,14]. After IRI, HA deposition in submucosal ganglia increased, mirroring previous data on IRI-induced HA accumulation both in peripheral tissues and in the CNS [47,48], and depends upon a 4-MU-sensitive HAS2 upregulation [7,47]. Increased HA levels in the submucosal plexus may impact the excitability of secretomotor, vasomotor and intrinsic sensory neurons connecting the submucosal plexus with myenteric ganglia and involved in motor reflexes [49].

After IRI, smooth muscle cells displayed signs of damage, showing cytoplasmic vacuolization and presence of spaces between cells. Such changes were not observed after sham operation, suggesting that IRI-induced damage to smooth muscle cells surpasses laparotomy and intestine handling. After IRI, alterations of the *muscularis propria* may depend upon development of pro-inflammatory conditions, as suggested by the highest degree of neutrophilic infiltration [4,41]. Interestingly, IRI-induced injury in the *muscularis propria* was also associated with the highest degree of IRI-mediated enhancement in HA deposition in the gut wall. IRI-induced HA levels and neutrophil number in the smooth muscle layer were drastically reduced after 4-MU treatment, suggesting that local mechanism/s involving HA homeostasis and function are sensitive to IRI injury. Importantly, in the sham-operated group, both parameters were not significantly influenced by 4-MU, suggesting a specific action of 4-MU on IRI-induced HA homeostasis changes in the smooth muscle layer. Myenteric neurons also displayed prominent signs of IRI-induced injury, although their total number did not change in this model, as previously demonstrated [7]. After IRI, HA deposition and the number of neutrophils increased in myenteric ganglia, and both parameters were drastically reduced by 4-MU treatment. Notably, in the 4-MU-treated injured group, myenteric neurons displayed minor signs of damage. Overall, these observations suggest that enhanced HA deposition in myenteric ganglia may influence both the neuronal and immune enteric functions. We cannot exclude that such modulation may favor a neuroimmune cross-talk, where neurons and neutrophils may influence one another’s function, possibly leading to long-lasting neuromuscular damage [50,51].

In the untreated and 4-MU-treated injured groups, metagenomic analysis did not reveal major intragroup changes in the composition of the fecal saprophytic bacterial community. However, in non-injured and sham-operated groups, 4-MU treatment influenced bacterial intragroup composition. In particular, the reduced variability observed in samples obtained from non-injured animals, after 4-MU treatment, underlines the relevance of HA in maintaining gut bacteria homeostasis in normal conditions [52]. HA may also influence commensal bacterial variability after laparotomy, as suggested by the ability of 4-MU to restore α-diversity indexes in the sham-operated group. No significant differences were also observed in the microbiota composition among the different groups, and, notably, in agreement with other studies [53], the composition of the gut microbiota in the injured group was not significantly different from the non-injured group.

At the phylum level, a significant enhancement of Proteobacteria was observed in the injured group, which was insensitive to 4-MU. Within Proteobacteria, the relative abundance of the genus *Escherichia*, which comprises potentially pathogenetic species, significantly increased after IRI. Increased levels of *Escherichia* spp. were shown to be an indicator of dysbiosis underlying IBD pathogenesis [46]. A reduction in Firmicutes was observed after IRI, which was insensitive to 4-MU. Within this phylum, the genus *Enterococcus*, which retains a potentially pathogenetic role in IBD, significantly increased, while *Lactobacillus* and *Clostridium*, which are known as mainly favorable bacteria, were reduced after IRI [46]. After IRI, changes in *Escherichia*, *Enterococcus*, *Lactobacillus* and *Clostridium* levels were abolished by 4-MU, suggesting that blockade of HA synthesis may have a favorable effect on microbiota–host interplay by reducing the presence of harmful bacteria and increasing that of beneficial bacteria. Laparotomy also promoted the growth of intestinal harmful bacteria, as suggested by the enhanced levels of *Escherichia* and *Enterococcus* after sham operation. However, both *Escherichia* and *Enterococcus* were less sensitive to 4-MU treatment in this experimental group, indicating that de novo synthesis of HA may be less relevant to gut microbial composition changes, in this experimental condition.

According to previous reports, in the rat small intestine, TLR2 and TLR4 were found in different cell types. In particular, both TLR2 and TLR4 immunoreactivity was detected in neurons and glial cells of the submucosal and myenteric plexus and in sparse inflammatory cells, suggesting that TLRs may participate in neuroimmune interaction [9,10,38]. After IRI, in good agreement with the highest degree of HA deposition enhancement and neutrophilic migration, TLR2 and TLR4 mRNA and protein levels more prominently increased in LMMP preparations than in the submucosal layer. The possible correlation between IRI-mediated changes in HA homeostasis and changes in TLR2 and TLR4 expression is strengthened by the evidence that 4-MU significantly reduced IRI-induced TLR2 and TLR4 upregulation, suggesting that HA de novo synthesis may influence TLR2 and TLR4 transcription and translation in this condition.

The consequences of TLR2 and TLR4 upregulation in the neuromuscular compartment during IRI were evaluated by studying the effect of the TLR2/4 antagonist, Sparstolonin B (SsnB), on the excitatory and inhibitory longitudinal muscle responses. SsnB is a polyphenolic natural compound that improved intracerebral hemorrhage and endotoxin shock outcomes in mice in a TLR2/TLR4-dependent manner [39,54].

After IRI, the contractile response to CCh was significantly lower compared to the non-injured and sham-operated group, as expected, suggesting that a transitory ischemic injury to the rat small intestine followed by reperfusion hampers muscarinic post junctional contractile responses [7,55]. In our previous investigation, however, IRI-induced impairment of CCh-stimulated longitudinal muscle contractions was not influenced by 4-MU, indicating that changes in HA deposition after IRI do not influence post junctional cholinergic responses [7]. In all experimental groups, SsnB concentration-dependently reduced the maximal effect of the concentration–response curve to CCh, but did not influence the potency (EC_50_), suggesting that TLR2 and TLR4, located on smooth muscle cells, may exert a tonic facilitation of post junctional excitatory responses, as already suggested by other studies [38,56]. In the injured and sham-operated groups, SsnB was more effective in downregulating the curve to CCh with respect to non-injured animals. Such sensitivity to SsnB does not seem to depend upon changes in TLR2/4 expression, at least in the sham-operated group. After IRI, EFS-induced contractions of neuronal origin were reduced, as expected [7,57], and may depend upon neosynthesized HA, because administration of 4-MU significantly increased EFS-evoked responses, in this condition [7]. In the injured group, but not in the non-injured and sham-operated groups, contractions at 5 and 10 Hz were significantly reduced by SsnB in a concentration-dependent manner, indicating that TLR2/4 activation contributes to the prejunctional component of the excitatory contraction. The prominent HA-mediated upregulation of TLR2 and TLR4 receptor expression in LMMPs may explain the sensitivity of EFS-induced contractions to SsnB after IRI. In the peristaltic reflex, excitatory neuronal pathways may be particularly sensitive to hypoxic/ischemic injury, which may damage cholinergic neuronal pathways as well as other neurotransmitters, such as tachykinins [55,57]. However, in the injured group, non-adrenergic non-cholinergic (NANC) off-contractions, which are tachykinin-mediated, were insensitive to SsnB, suggesting that TLR2/4 may preferentially affect cholinergic pathways.

NANC on-relaxations were reduced in the injured group as expected, suggesting that, also in our model, IRI significantly alters the inhibitory component of the peristalsis, as also demonstrated by other authors [55]. Inhibition of NANC on-relaxations during IRI does not depend upon IRI-induced HA de novo synthesis since it is not influenced by 4-MU administration [7]. On the other hand, the present data suggest that HA may sustain relaxation responses after IRI via TLR2/4 upregulation, since NANC on-relaxations were downregulated by SsnB in the IRI, but not in the non-injured and sham-operated groups. Interestingly, in the injured group, SsnB did not influence longitudinal muscle relaxations in the presence of L-NAME, suggesting that TLR2/4 activation modulates the nitrergic component of relaxation, more than other inhibitory enteric neurotransmitter pathways, in this condition [58].

## 5. Conclusions

In the present study, we demonstrate that, after IRI, endogenous HA may participate in the development of a proinflammatory condition by favoring microbial dysbiosis and neutrophilic recruitment, which may contribute to long-lasting neuromuscular changes. These effects are, at least provisionally, compensated by HA-induced upregulation of TLR2 and TLR4. Increased TLR2/4 engagement facilitates both the excitatory and inhibitory component of the peristaltic reflex, aiding a mechanistic explanation to our previous demonstration of the beneficial effect of endogenous HA on the intestinal transit after IRI [5]. A more detailed evaluation of the different mechanisms underlaying HA deposition and fragmentation during intestinal IRI injury is required to better understand HA involvement in disease-related gut microbiota–neuroimmune axis changes, thus potentially fostering new therapeutic approaches.

## Figures and Tables

**Table 1:** List of primary and secondary antisera.

**Primary Antibody**	**Host Species**	**Dilution** **(HC)**	**Dilution** **(WB)**	**Specificity**	**Source**
HuC/D biotin	Mouse	1:100	_	Neuronal cells [27]	Invitrogen (A-21272)
HABP	_	1:100	_	Hyaluronan [28]	Hokudo (BC41)
HAS2	Goat	1:100	_	HAS2 [29]	Santa Cruz Biotechnology (sc-34068)
MPO	Rabbit	1:1		Myeloperoxidase [25]	Dako (GA511)
TLR2	Rabbit	1:500	1:1000	TLR2 [30]	ABclonal (A11225)
TLR4	Rabbit	1:800	1:1000	TLR4 [31]	ABclonal (A5258)
β-actin	Mouse	_	1:1000	Housekeeping [32]	Cell Signaling (#3700)
**Secondary Antibody** **and Streptavidin complex**					
Anti-rabbit IgG HRP-linked	Donkey	_	1:5000		Amersham (NA934)
Anti-mouse IgG HRP-linked	Horse	_	1:2000		Cell Signaling (7076S)
Anti-rabbit IgG, biotin	Goat	1:200	_		Abnova Corporation (PAB10824)
Anti-goat IgG, biotin	Rabbit	1:200	_		Abnova Corporation (PAB10578)
Anti-goat IgG Cy3-conjugated	Donkey	1:500	_		Jackson Immuno Research Laboratories
FITC-Streptavidin conjugated		1:200	_		Molecular Probes (SA1001)
Cy3-Streptavidin conjugated		1:500	_		Amersham (PA43001)

**Table 2:** Primer sequences.

Gene	Sequence
β-actin	F 5′-TGACAGGATGCAGAAGGAGA-3′ R 5′-TAGAGCCACCAATCCACACA-3′
TLR2	F 5′-CCGAAACCTCAGACAAAGCG-3′ R 5′-ACAGCGTTTGCTGAAGAGGA-3′
TLR4	F 5′-TGAGATTGCTCAAACATGGC-3′ R 5′-CGAGGCTTTTCCATCCAATA-3′

## Data Availability

The data presented in this study are available on request from the corresponding author.

## Supplementary Materials

The following supporting information can be downloaded at: https://www.mdpi.com/article/10.3390/cells11213370/s1. Supplementary Figure S1: Frequency and amplitude of the spontaneous contractions in the rat small intestine longitudinal muscle recorded in vitro. Table S1 Morphology parameters of the rat small intestine. Table S2: Effect of Sparstolonin B on EC_50_ values of carbachol concentration–response curves.
