# Supplementary material for: Hyaluronan Regulates Neuronal and Immune Function in the Rat Small Intestine and Colonic Microbiota after Ischemic/Reperfusion Injury
Source: Cells. 2022 Oct 25;11(21):3370. doi: 10.3390/cells11213370 (PMC9657036; doi:10.3390/cells11213370)
Supplement: Supplementary file 1 [file cells-11-03370-s001.zip › cells-1848683-supplementary.pdf]

Supplementary Table S1

Table S1. Morphology parameters of the rat small intestine.

Parameter	non-injured	non-injured 4-MU	Sham	Sham 4-MU	injured	injured 4-MU
Mucosa thickness (μm)	645.90 ± 21.36	578.90 ± 16.28	665.60 ± 7.94	592.4 ± 18.85	554.50 ± 15.51**,°°°	612.30 ± 9.83
Villi Height (μm)	381.6 ± 7.086	361.9±15.94	394.9 ± 9.79	351.30 ± 16.84	322.10 ± 10.82**,°°°	372.30 ± 13.42
Villi width (μm)	71.67 ± 2.76	75.29 ± 3.47	74.19 ± 2.18	67.14 ± 2.78	67.81 ± 2.17	69.96 ± 1.66
Villi density (no of villi/ 0.5 mm linear mucosa)	2.75 ± 0.11	3.42 ± 0.17	2.99 ± 0.14	3.57 ± 0.28	4.00 ± 0.20**,°°	3.32 ± 0.21
Crypt depth (μm)	264.2 ± 14.87	217.8 ± 16.28	258.5 ± 3.42	241.1±18.85	232.4 ± 6.59	240 ± 9.83
Submucosa thickness (μm)	26.44 ± 1.56	29.53 ± 3.25	30.84 ± 1.34	27.29 ± 1.62	27.23 ± 0.92	28.36 ± 1.36
Circular muscle thickness (μm)	62.26 ± 1.83	63.37 ± 5.03	61.36 ± 2.17	58.05 ± 1.97	66.28 ± 2.34	62.58 ± 4.47
Longitudinal muscle thickness (μm)	31.76 ± 2.04	30.19 ± 3.44	33.31 ± 1.51	32.72 ± 1.89	30.48 ± 1.27	31.59 ± 2.33
Paneth cell number	73.63 ± 9.74	44.25 ± 3.11**	67.25 ± 6.27	42.0 ± 5.07	64.63 ± 4.04	47.25 ± 5.76
Goblet cell number	333.3 ± 47.65	368.3 ± 34.51	325.8 ± 16.74	352.7 ± 33.92	379.6 ± 25.81	282.0 ± 15.87

Values are presented as the mean ± SEM; N=5 rats/group. ** = P<0.01 vs non-injured; °° = P<0.01 and P = <0.001 vs Sham by one-way ANOVA with Tukey's test.

Supplementary Table S2

Table S2. Effect of Sparstolonin B on EC_{50} values of carbachol concentration–response curves

Experimental group	EC_{50} (μM) with 95% CI
non-injured	0.14 (0.08-0.25)
non-injured SSnB 10 μM	0.16 (0.11-0.26)
non-injured SSnB 30 μM	0.17 (0.09-0.28)
sham	0.15 (0.11-0.19)
sham SSnB 10 μM	0.25 (0.14-0.49)
sham SSnB 30 μM	0.35 (0.13-1.69)
injured	0.13 (0.06-0.27)
injured SSnB 10 μM	0.29 (0.18-0.48)
injured SSnB 30 μM	0.48 (0.26-0.97)

Values are the mean ± SEM; *n* = 5 rats/group. No significant differences were observed in EC_{50} values among all groups by one-way ANOVA with Tukey's post hoc test. CI: confidence interval.

Supplementary Figure S1

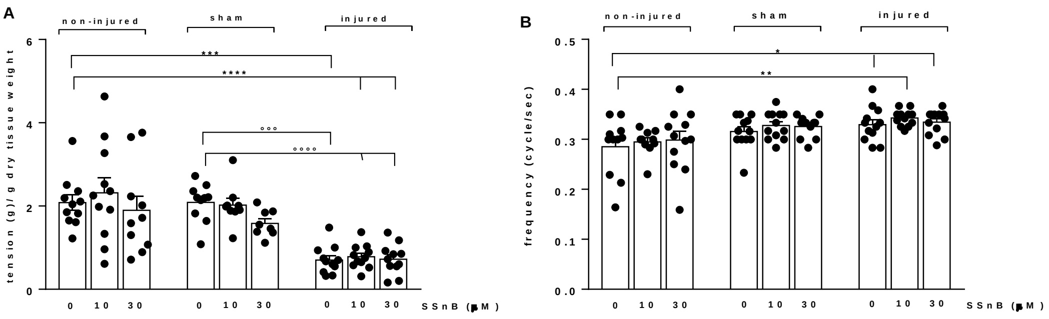

Supplementary Figure S1. Frequency and amplitude of the spontaneous contractions in the rat small intestine longitudinal muscle recorded in vitro. **(A)** Amplitude values are expressed as gram of tension. **(B)** Frequency values are expressed as cycle/sec.Values are the mean ± SEM. ****,°°°° = P<0.0001, ***,°°° = *P*< 0.001 ** = *P*< 0.01 * = *P*< 0.05 by one-way ANOVA followed by Tukey's post hoc test. .
